# Source of Oxygen Fed to Adventitious Roots of *Syzygium kunstleri* (King) Bahadur and R.C. Gaur Grown in Hypoxic Conditions

**DOI:** 10.3390/plants9111433

**Published:** 2020-10-24

**Authors:** Hong-Duck Sou, Masaya Masumori, Goro Ezaki, Takeshi Tange

**Affiliations:** 1 Urban Forests Research Center, National Institute of Forest Science, Seoul 02455, Korea; 2 Graduate School of Agricultural and Life Sciences, The University of Tokyo, Yayoi 1-1-1, Bunkyo-ku, Tokyo 113-8657, Japan; masumori@fr.a.u-tokyo.ac.jp (M.M.); goro_ezaki160@maff.go.jp (G.E.); tangetakeshi@g.ecc.u-tokyo.ac.jp (T.T.)

**Keywords:** adventitious roots, hypoxic conditions, oxygen transportation, water level elevation, woody plants, *Syzygium kunstleri*

## Abstract

*Syzygium kunstleri*, a woody plant species, adapts to hypoxic conditions by developing new adventitious roots. Here, we investigate its morphological adaptation to long-term water level changes and the sources and pathways of O_2_ supplied to its adventitious roots. Cuttings were cultivated in hydroponic and agar media, and then, the water level was increased by 6 cm following adventitious root emergence; afterward, O_2_ partial pressure changes were measured using a Clark-type O_2_ microelectrode. O_2_ concentrations in the adventitious roots decreased when N_2_ was injected, regardless of the presence of light, indicating that the O_2_ source was not photosynthetic when bark was removed. New adventitious roots developed near the surface when the water level increased, and O_2_ conditions above the raised water level influenced O_2_ concentrations in adventitious roots. O_2_ concentrations in adventitious roots that developed before the water level increased were lower than in the newly developed adventitious roots but increased when the O_2_ concentrations above the original water level increased. Our study highlights morphological changes, such as the development of adventitious roots, as environmental adaptation mechanisms. By revealing O_2_ sources in *S. kunstleri* under hypoxic environments, we offer insights into the challenges of long-term adaptation to changing environments in woody plants.

## 1. Introduction

Adaptation to flooding is generally related to the ability to overcome the limited oxygen (O_2_) supply to roots, as plants become exposed to the flooded conditions and O_2_ deficiency occurs [1]. A flood-tolerant plant can overcome the adverse effects of flooding through various morphological adaptations, such as hyponasty (upward bending of leaves), enhanced shoot elongation, aerenchyma formation, the development of barriers against radial O_2_ loss (ROL) in roots, the development of adventitious roots, modifications of the leaf anatomy, and the formation of gas film on leaf surfaces [1,2,3,4,5,6,7,8]. Adventitious roots help plants adapt to flooding, effectively transport atmospheric O_2_ into the roots, and may support or replace the primary root system [7]. Additionally, aerenchyma occur in adventitious roots and act as an O_2_ transportation pathway.

The morphological adaptation to the hypoxic environment supports O_2_ transport to roots developed in the submerged portion. The development of secondary aerenchyma promotes large cracks (hypertrophic lenticels) in the stems and roots, exposing the secondary aerenchyma to the atmosphere through lenticels, thereby promoting the uptake of atmospheric O_2_ [9]. In *Oryza sativa* and *Halosarcia pergranulata*, O_2_ in aerenchymatous roots decreased when photosynthetic tissue was restricted by light [10,11]. Underwater photosynthesis is also known to generate O_2_ that functions as an internal O_2_ source for root oxygenation [12]. 

Changes in water levels are thought to influence the source and pathway of O_2_ supplied to the roots. Usually, in the tropics, the wet season lasts from May to October and is influenced by the southwest monsoon. Thus, some plants are exposed to hypoxic conditions for long periods of time. It has been found that short-term changes in water levels cause changes in the O_2_ partial pressure (pO_2_) of the root and that O_2_ in the atmosphere is exchanged into the roots [13]. However, for long-term changes in water levels, there is limited information on the source of the O_2_ in adventitious roots and the pathway through the stem into the adventitious roots. Based on the fact that *Syzygium kunstleri* grows during the long-term, increased water level during the rainy season, we hypothesized that the O_2_ concentration in the roots of *S. kunstleri* is maintained, even during long-term exposure to a hypoxic environment. In this study, we investigated the morphological adaptations of *S. kunstleri* to water level changes and the source and pathway of O_2_ supplied to the subsequently developed adventitious roots.

## 2. Results

### 2.1. Effect of Light Changes on the Internal pO_2_ in Adventitious Roots

**Figure 1:**
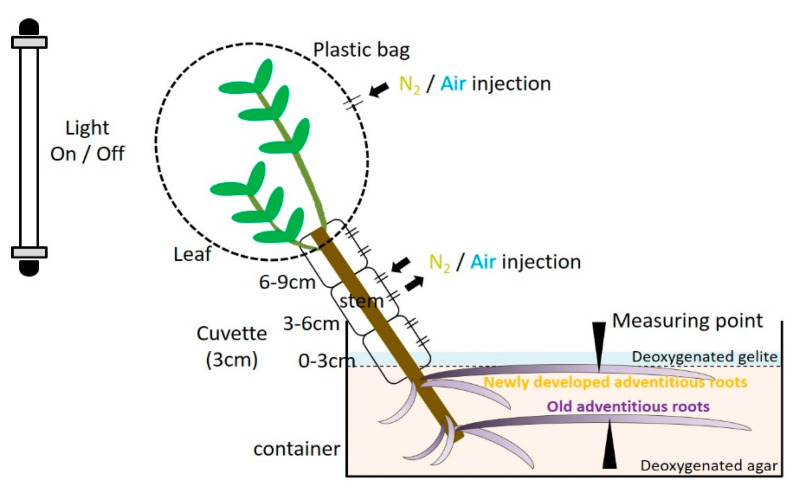
Diagram of the experiment. Air and N_2_ injected 0–3, 3–6, and 6–9 cm above the water surface(arrows). A plastic bag was attached to restrict oxygen to each part of the stem and leaf. The root system was embedded in deoxygenated agar to restrict oxygen. Changes in the oxygen partial pressure (pO_2_) were measured using a Clark-type O_2_ microelectrode(arrowheads) in adventitious roots.

**Figure 2:**
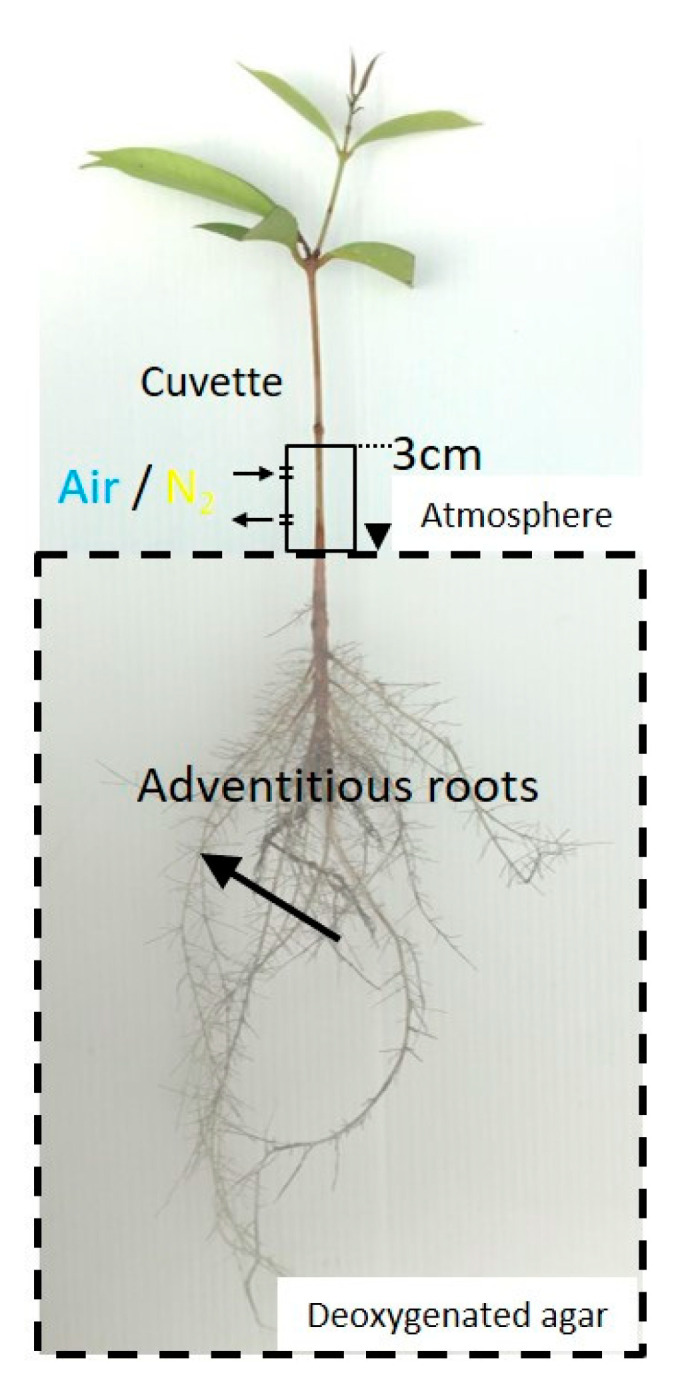
The effect of illumination on the oxygen partial pressure (pO_2_) in adventitious roots of *Syzygium kunstleri*. The light condition was controlled during the injection of air or N_2_ in the stem through the cuvette(arrows), 0–3 cm above the water surface. Light and dark conditions were alternately changed at intervals of 30 min. The root system was fixed with deoxygenated solid agar (1.5 % *w/v*). Arrow: measurement point; arrowhead: water level.

**Figure 3:**
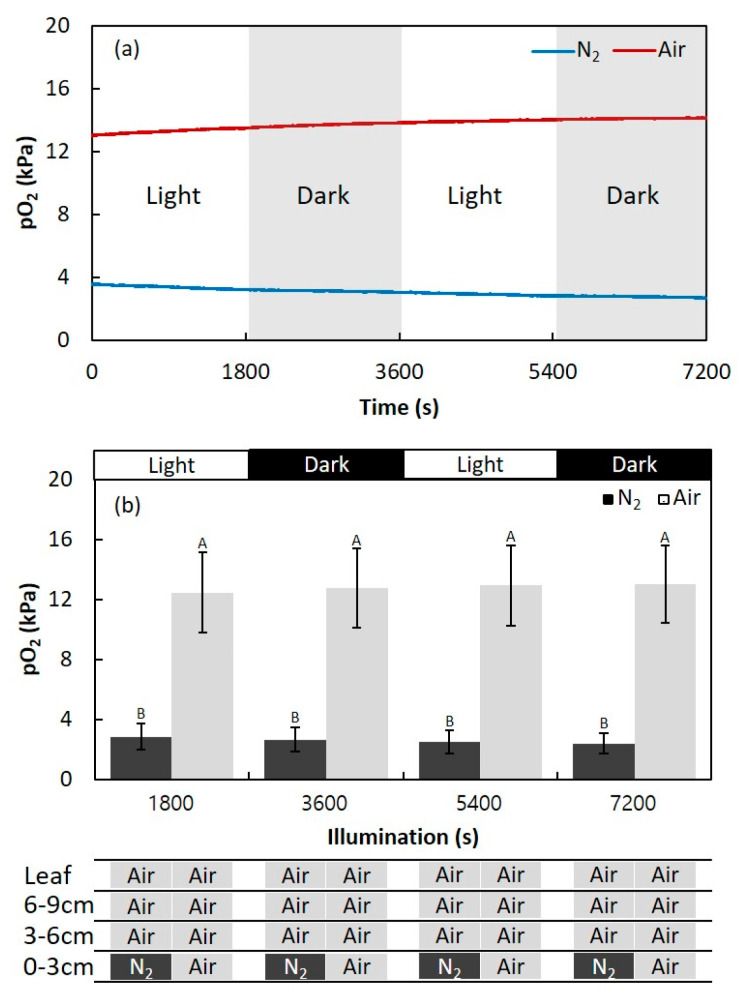
The effect of illumination on the oxygen partial pressure (pO_2_) in adventitious roots of *Syzygium kunstleri*. (**a**) Time-trace of pO_2_ in adventitious roots during N_2_ and air injection with the light conditions controlled. (**b**) The light condition was controlled during injection of air or N_2_ into the 0–3-cm section of the stem. Light and dark conditions were alternately changed at intervals of 30 min. The root system was fixed with deoxygenated solid agar (1.5 % *w/v*). Values are means ± SD. (*n* = 6 roots from 6 individual plants, average of the last one min before changing treatments). The same letter means no significant difference within the same area (*p* < 0.05, one-way ANOVA, followed by Tukey’s test for multiple comparisons).

The dynamics of pO_2_ within the adventitious roots that developed in hypoxic conditions were measured in the cortex using an O_2_ microelectrode, positioned 10 mm above the root–shoot junction, while light and dark conditions were alternately changed at intervals of 30 min (Figure 1 and Figure 2). When air was injected, root pO_2_ was maintained at about 13 kPa and did not show any response to alternating light conditions. In contrast, when N_2_ was injected, root pO_2_ was maintained at about 3 kPa. Thus, while the stem was exposed to air or N_2_, the root pO_2_ was not significantly affected by the changes in illumination (Figure 3).

### 2.2. Secondary Aerenchyma Functioning as Oxygen Pathway in the Stem

**Figure 4:**
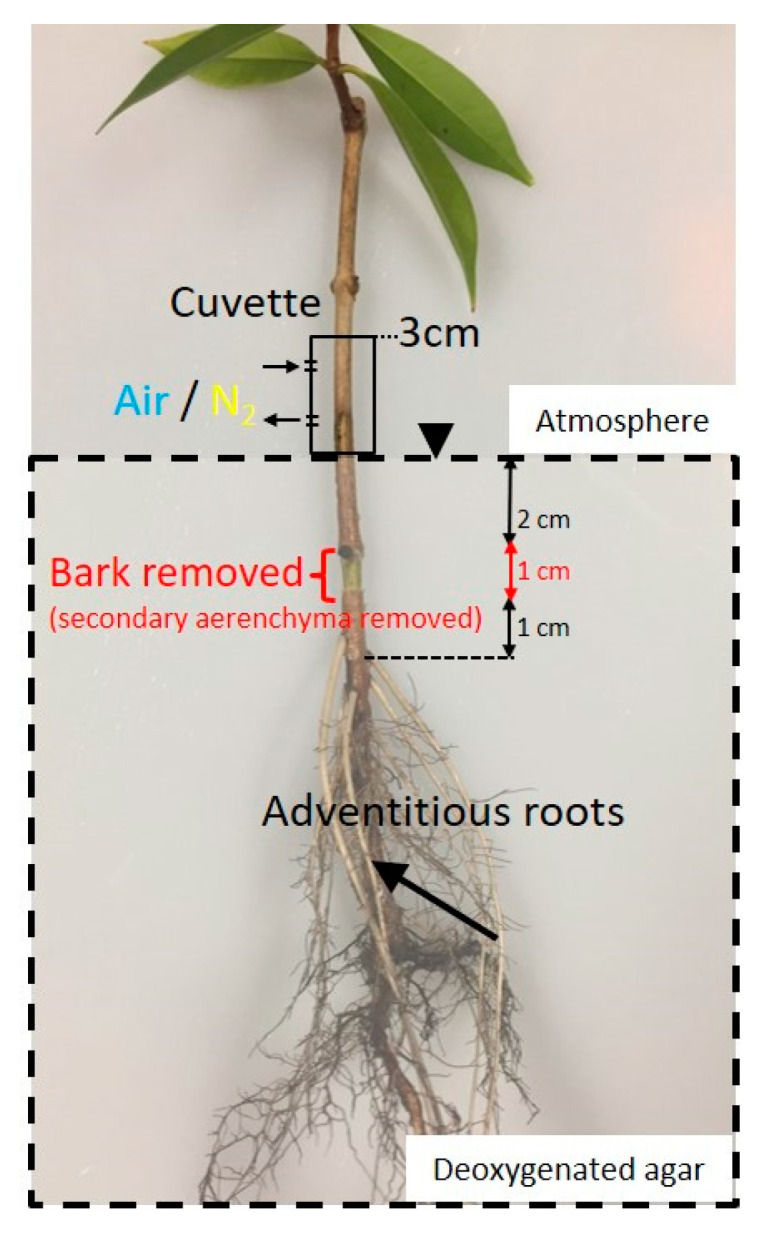
The effect of removal of secondary aerenchyma developed in the stem on the oxygen partial pressure (pO_2_) in the adventitious roots of *Syzygium kunstleri*. A 1 cm long section of bark (secondary aerenchyma) was removed from the stem, located 2 cm below the water surface. Air or N_2_ was injected at the 0–3 cm portion of stem above the water level through the cuvette(arrows). Arrow: measurement point; arrowhead: water level.

**Figure 5:**
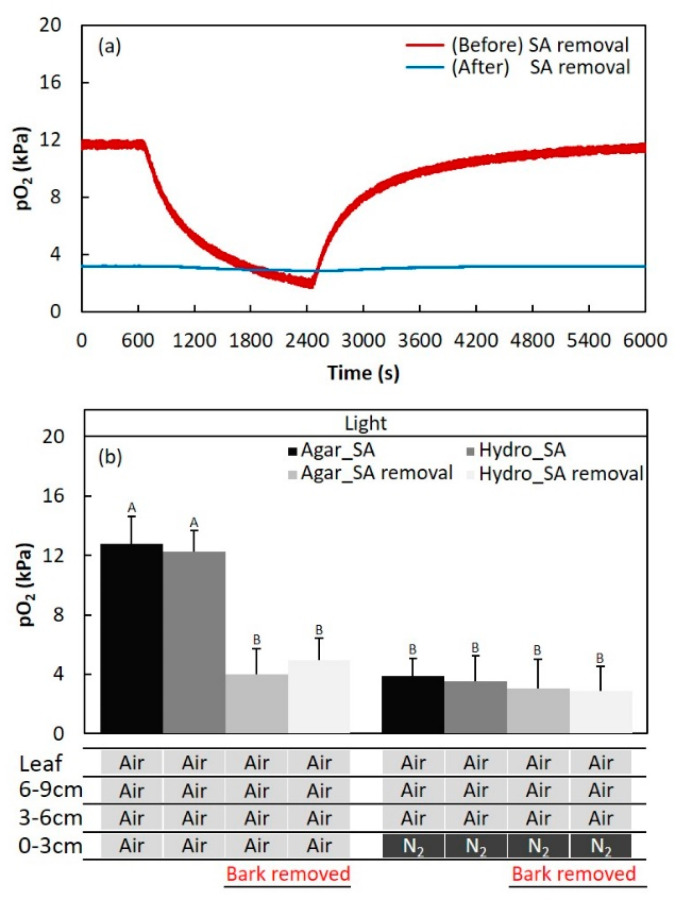
The effect of removal of secondary aerenchyma (SA) developed in the stem on the oxygen partial pressure (pO_2_) in the adventitious roots of *Syzygium kunstleri* grown on hydroponic and agar media. (**a**) Time-trace of pO_2_ in the adventitious roots before and after the removal of SA in the stem. N_2_ was injected in exchange of air from 600 to 2400 s at the 0–3 cm-section of the stem above the water level. (**b**) A 1-cm-long section of bark (SA) was removed from the stem, located 2 cm below the water surface. Air or N_2_ was injected at the 0–3-cm portion of the stem above the water level before and after SA removal. Values are means ± SD. (*n* = 6 roots from 6 individual plants, average of the last one minute before changing the treatments). The same letter means no significant difference within the same area (*p* < 0.05, one-way ANOVA, followed by Tukey’s test for multiple comparisons). Agar: agar medium, Hydro: hydroponic medium, and SA: secondary aerenchyma.

The secondary aerenchyma in the stem, 2 cm below the water level, was removed, and the dynamics of pO_2_ within the roots were measured using an O_2_ microelectrode, positioned 10 mm from the root–shoot junction of the adventitious roots (Figure 4). Air or N_2_ was injected into the stem, 0–3 cm above the water level. Before the removal of secondary aerenchyma in the stem, the internal pO_2_ within the root was about 12–13 kPa when air was injected into the stem. After the removal of secondary aerenchyma in the stem, the internal pO_2_ within the root decreased to about 4–5 kPa when air was injected into the stem. Regardless of the presence or absence of secondary aerenchyma in the stem, the internal pO_2_ within the root was 2–4 kPa when N_2_ was injected into the stem. After the secondary aerenchyma developed in the stem was removed, the pO_2_ in the adventitious roots did not respond to the injection of air or N_2_ (Figure 5). This indicates that the secondary aerenchyma in the stem functions as an O_2_ transport pathway from the stem to the roots.

### 2.3. Entry Portion of Atmospheric Oxygen in the Stem

**Figure 6:**
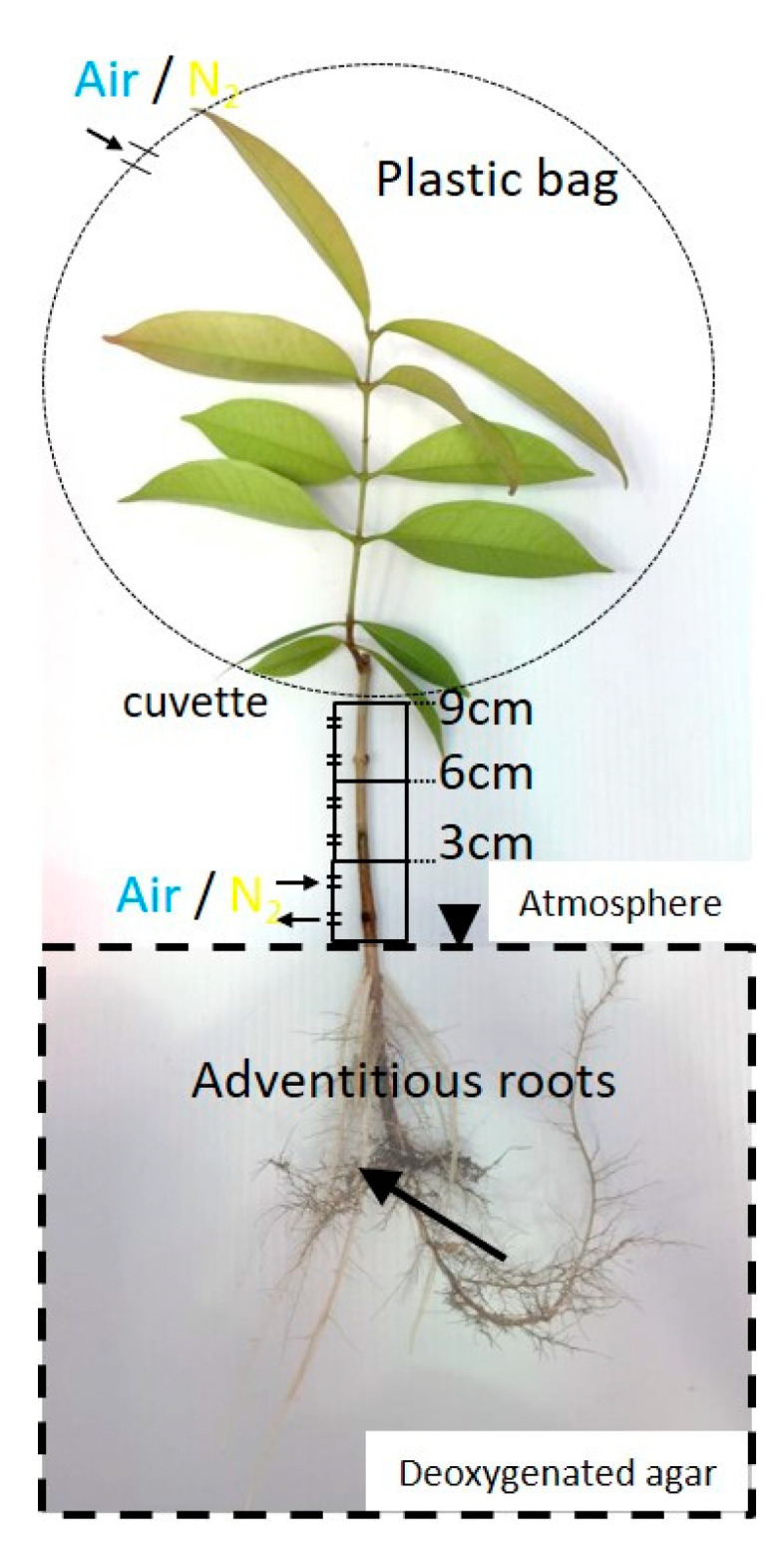
Entry portion of atmospheric oxygen into the stem of *Syzygium kunstleri*. Air or N_2_ was injected through a 3-cm-cuvette and plastic bag to restrict oxygen from each part of the stem and leaf. The stem was divided into four parts: 0–3 cm, 3–6 cm, and 6–9 cm above the water level and the leaf. N_2_ was injected for 30 min through the cuvette covering the stem and for 1 h through a plastic bag-sealed leaf(arrows). Arrow: measurement point; arrowhead: water level.

The position of the cuvette was changed in four parts (0–3 cm, 3–6 cm, and 6–9 cm above the water level and the leaf), and air or N_2_ was injected into each part to control the O_2_ conditions (Figure 1 and Figure 6). The dynamics of pO_2_ within the adventitious roots were then measured. 

**Figure 7:**
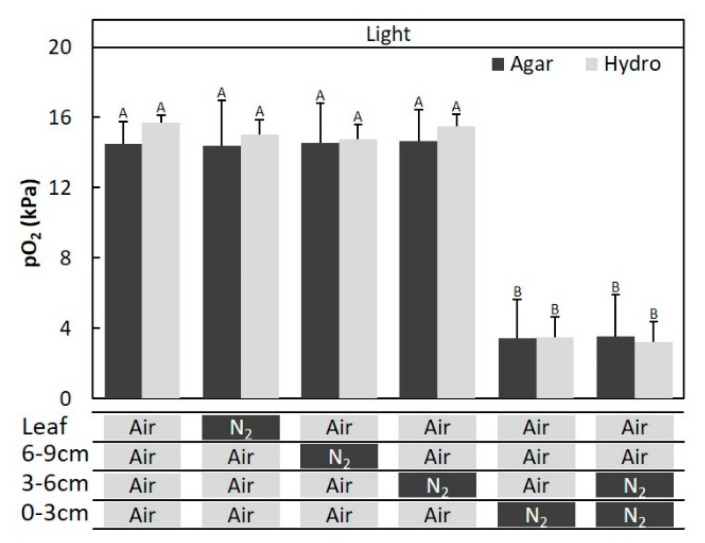
Entry portion of atmospheric oxygen in the stem of *Syzygium kunstleri* grown on hydroponic or agar media. Air or N_2_ was injected into each part of the stem (0–3 cm, 3–6 cm, and 6–9 cm above the water level) and the leaf. N_2_ was injected for 30 min through the cuvette covering the stem and for 1 h through a plastic bag-sealed leaf. Values are means ± SD. (*n* = 6 roots from 6 individual plants, average of the last minute before changing the treatments). The same letter means no significant difference within the same area (*p* < 0.05, Bonferroni corrected Mann–Whitney pairwise comparisons). Agar: agar medium and Hydro: hydroponic medium.

The injection of air or N_2_ at the 3–6-cm or 6–9-cm portions of the stem did not induce any significant changes in the pO_2_ within the adventitious roots (Figure 7). In addition, air or N_2_ injection through the plastic bag to the leaf part did not induce a significant change in the pO_2_ in the adventitious roots (Figure 7). Only the N_2_ injection in the stem at the 0–3-cm portion decreased the pO_2_ in roots. The root pO_2_ was changed significantly only when the environmental conditions in the 0–3-cm portion were changed (Figure 7).

### 2.4. Entry Portion of Atmospheric Oxygen in the Stem after Water Level Elevation

**Figure 8:**
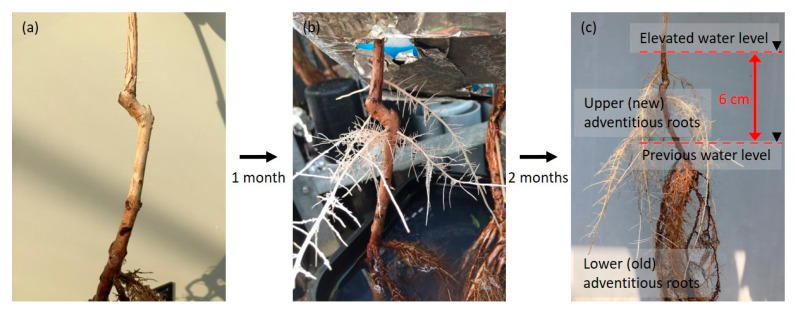
The treatment of the water level elevation, and the development of adventitious roots in *Syzygium kunstleri*. (**a**) Before the water level elevation treatment. (**b**) One month after water the level elevation treatment. (**c**) Three months after the water level elevation treatment. The water level was successively increased by about 6 cm. New adventitious roots developed near the elevated water surface. Arrowhead: water level.

**Figure 9:**
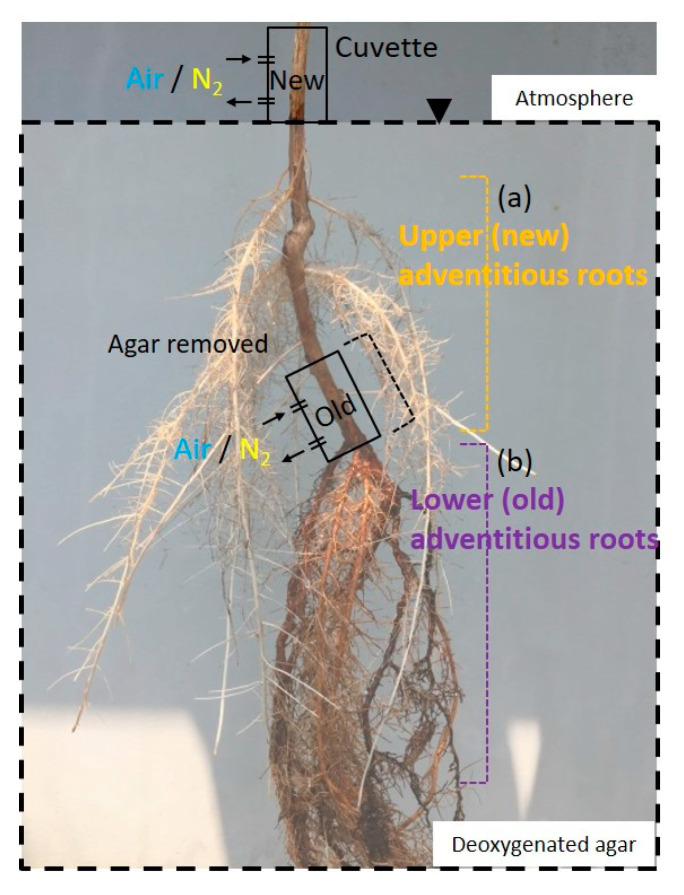
Entry portion of atmospheric oxygen in the stem of *Syzygium kunstleri* after water level elevation. (**a**) Upper (new) adventitious roots, which developed after the water level elevation treatment. (**b**) Lower (old) adventitious roots, which developed in the flooding treatment. New cuvette positioned at the 0–3-cm portion of the stem based on the elevated water level. Old cuvette positioned at the 0–3-cm portion of the stem based on the prior water level. Air and N_2_ were injected through the cuvette(arrows). Arrowhead: water level.

After raising the water level up to 6 cm from the initial level, new adventitious roots developed on the stems near the elevated water surface (Figure 8). Air or N_2_ was injected into the stem at each of the four points: 0–3 cm, 3–6 cm, and 6–9 cm above the elevated water level and the leaf level. Thereafter, the dynamics of the pO_2_ in the upper adventitious roots were measured (Figure 1 and Figure 9). 

**Figure 10:**
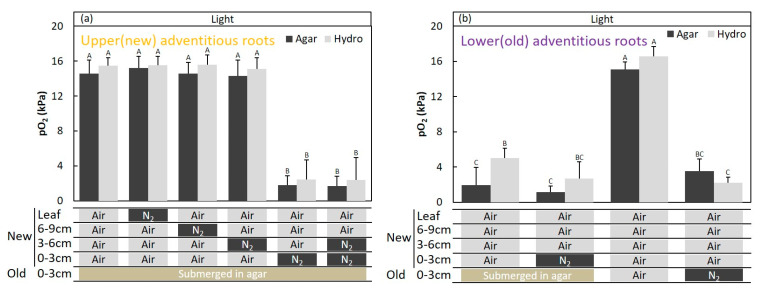
Entry portion of atmospheric oxygen in the stem of *Syzygium kunstleri* after water level elevation. The pO_2_ was measured in (**a**) the upper (new) adventitious roots and (**b**) lower (old) adventitious roots, which developed after the water level elevation treatment. (**a**) Cuvette positioned at the 0–3-cm portion of the stem based on the elevated water level. Air or N_2_ was injected into each part of the stem (0–3 cm, 3–6 cm, and 6–9 cm from the elevated water level) and the leaf. N_2_ was injected for 30 min through the cuvette covering the stem and for 1 h through a plastic bag-sealed leaf. Values are means ± SD. (*n* = 6 roots from 6 individual plants, average of the last one minute before changing the treatments). The same letter means no significant difference within the same area (*p* < 0.05, Bonferroni corrected Mann–Whitney pairwise comparisons). (**b**) The cuvette was positioned at the 0–3-cm portion of the stem based on the elevated water level. The cuvette was positioned at the 0–3-cm portion of the stem based on the prior water level after the agar medium was removed. N_2_ or air was injected into the old or new cuvette position. Values are means ± SD. (*n* = 6 roots from 6 individual plants, average of the last minute before changing the treatments). The same letter means no significant difference within the same area (*p* < 0.05, one-way ANOVA, followed by Tukey’s test for multiple comparisons). Agar: agar medium and Hydro: hydroponic medium.

The injection of air or N_2_ into the 3–6-cm or 6–9-cm stem portions did not have a significant effect on the changes in the pO_2_ within the new adventitious roots (Figure 10a), and the injection of air or N_2_ into the leaf through the plastic bag did not induce a significant change in the pO_2_ in the new adventitious roots (Figure 10a). N_2_ injection at 0–3 cm above the water level only caused a significant change in the pO_2_ in the upper adventitious roots. The root pO_2_ was significantly influenced by the O_2_ condition at the 0–3-cm portion of the stem, based on the water levels in the upper adventitious roots (Figure 10a). The results of “N_2_ in 0–3 cm and Air in 3–6 cm” and “N_2_ in 0–3 cm and 3–6 cm” show that the entry portion of O_2_ is limited to the 0–3-cm portion of the stem (Figure 10a).

Changes in the pO_2_ in the lower adventitious roots were measured after fixing the root system in a deoxygenated solid agar (1.5%) medium, based on the elevated water levels. Air or N_2_ was injected into the 0–3-cm part of the stem based on the elevated water level, while the prior 0–3 cm of the stem portion was submerged in agar media (Figure 1 and Figure 9). During air injection, the pO_2_ in the lower adventitious roots was 2–5 kPa, which was significantly lower than the pO_2_ in the upper adventitious roots (14 to 15 kPa; Figure 10b). When N_2_ was injected, the pO_2_ within lower adventitious roots was in the range of 2 to 3 kPa (Figure 10b). After the agar around the stem positioned at 0–3 cm above the initial water level was removed, air or N_2_ was injected into that area. When air was injected, the pO_2_ level within the lower adventitious roots was increased to 15 to 16 kPa (Figure 10b). The root pO_2_ was decreased to about 2 to 3 kPa when N_2_ was injected at the same position (Figure 10b).

## 3. Discussion

Exposing the shoot to the atmosphere or performing underwater photosynthesis is essential for flood-tolerant plants, because this enables internal aeration for O_2_-deficient tissues [14]. This study investigated the source and the pathway of O_2_ supplied to the adventitious roots in hypoxic conditions by measurement of the root pO_2_ in *S. kunstleri*. 

Some plants exposed to hypoxic conditions can improve their survival when carbohydrates are supplemented through photosynthesis [4]. In addition, underwater photosynthesis is known to produce O_2_ that functions as an internal O_2_ source for the aerobic respiration of submerged plants [12]. In *Oryza sativa* and *Halosarcia pergranlata*, the O_2_ in aerenchymatous roots clearly decreases when photosynthetic tissues are in the dark [5,10]. Additionally, in *Rumex palustris* and *R. acetosa*, the root pO_2_ was elevated in submerged conditions with light [12]. In alder, root aeration was sustained by convective gas flow driven by above-ambient pressures in the stem, arising from stem warming and the resulting thermal osmosis [15]. In *Salix martiana* and *Tabernaemontana juruana*, however, flooding of up to 0.5–1 mm and 1 to 2 cm above the origin of the root with O_2_-free water caused a rapid decline in the cortical O_2_ concentration [13]. Thus, in both species, experiments indicated that the internal O_2_ transport in roots does not depend on O_2_ generated by photosynthesis. The results of this study showed that the pO_2_ within the adventitious roots did not change even in the presence of light (Figure 3). Thus, in *S. kunstleri*, which is less affected by photosynthesis, contact with the atmosphere is important. Although not considered in this study, temperature gradients are known to affect gas transport from stem to root by thermo-osmotic pressure in the air space system [15]. 

The removal of secondary aerenchyma from flooded stems induced a decrease in the root pO_2_, which indicates that the secondary aerenchyma, developed in the stem, acts as an O_2_ transportation pathway (Figure 5). As observed in this study, it is known that the development of secondary aerenchyma in the stem enhances the transportation of atmospheric O_2_ into roots in hypoxic conditions. When the hypertrophic stem lenticels were blocked in soybeans, plant growth and root nodule activity were restricted [16]. As a result, hypertrophic lenticels were identified as entry portions of O_2_ into the aerenchyma [16]. In addition, when the hypertrophic lenticels were completely submerged, the pO_2_ in the aerenchyma developed in the stem gradually decreased [17]. Cracks caused by the development of secondary aerenchyma, namely hypertrophic stem lenticels, are the entry portions for O_2_ into the aerenchyma. In soybeans, secondary aerenchyma is developed as the tissue formed by the cell division of the pericycle arranged in the endodermis radially [18]. In *S. kunstleri*, however, cell division and several suberized cell layers are observed. Repeated occurrences of these suberized cell layers and the cell layer between them form a secondary aerenchyma [19]. A similar pattern was also found in *Lythrum salicaria* [20]. Secondary aerenchyma developed in *S. kunstleri* is thought to have a relatively lower porosity than that developed in soybeans [19]. More studies evaluating the transport of O_2_ in the secondary aerenchyma with these anatomical characteristics are needed.

The development of adventitious roots is a common response of plants to flooding [21]. In this study, adventitious roots began to develop in the stems of *S. kunstleri* after one to two weeks of flooding treatment. To overcome the stress caused by O_2_ deficiency in the root system, adventitious roots develop near the water surface or above the water surface in the stem (Figure 7 and Figure 8). For *S. kunstleri*, the first flooding treatment resulted in adventitious root development near the water surface, and the new adventitious roots formed again near the elevated water level afterward (Figure 8 and Figure 9). This result can be regarded as an adaptation to an efficient supply of O_2_ in the roots when the water level changes. According to Sauter [7], the formation of adventitious roots minimizes the distance for O_2_ diffusion and, thus, improves the O_2_ transportation into the roots under flooded conditions. In this study, each entry portion of O_2_ to old and new adventitious roots appeared to be different (Figure 7 and Figure 10). Therefore, it is considered that the main O_2_ entry portions of the old roots do not change after a rise in the water level but maintain the connection with the aerenchyma in the old roots. However, to clarify the transfer of O_2_ to the old roots, the development of secondary aerenchyma in the stem between the old adventitious roots and the new adventitious roots, as well as the potential transport of O_2_, should be confirmed. Additionally, it should be confirmed whether the old roots are alive. In *Alternanthera philoxeroides*, the adventitious roots developed in submergence can absorb O_2_ directly from the surrounding water to overcome the O_2_ deficiency [21]. Therefore, the O_2_ uptake capacity of the adventitious roots of *S. kunstleri* should also be considered. When O_2_ is transported from the atmosphere to the stem, O_2_ loss from the roots to the rhizosphere occurs because of the difference in concentrations. If it is difficult to absorb O_2_ from the atmosphere through the stem, the roots with lower O_2_ concentrations absorb O_2_ from the rhizosphere, where the O_2_ concentration is relatively higher. If the water surface remains elevated for a long period, newly developed roots near the water surface serve as the main roots. The existing roots, which do not have enough supply of O_2_ from the atmosphere, gradually weaken, and newly developed roots near the elevated water surface are thought to replace the functions of existing/old roots. This result confirms that the O_2_ supplied to the roots is believed to originate from the atmospheric air, near the elevated water surface. In conclusion, this study experimentally illustrates the source and pathway of O_2_ supplied to the adventitious roots of woody plants grown in hypoxic environments. This study will contribute to the understanding of the adaptation mechanisms to flooding in woody plants. 

## 4. Materials and Methods

### 4.1. Plant Materials

*Syzygium kunstleri* (King) Bahadur & R.C. Gaur was used for all experiments. Experiments were carried out with 6- to 12-month-old cuttings derived from 3- to 4-year-old saplings grown from seeds collected from a wetland in Narathiwat Province, Thailand. Cuttings were rooted in a pot (60 × 26 × 27 cm) containing Akadama soil (granular loamy soil) for about 6 months under well-watered conditions in a phytotron at the University of Tokyo, Japan. Conditions were maintained under a regime of 12 h of daylight at 30 °C (06:00–18:00), 12 h darkness at 25 °C (18:00–06:00), and a relative humidity of 60%–80%. After rooting, the cuttings were transferred to cylindrical pots (7 cm in diameter × 20 cm in height) containing quartz sand. Each pot held one cutting.

After washing the roots with tap water, the 10–15-cm-tall cuttings were transferred to either a hydroponic (control) or a stagnant agar medium in a container (17 × 25 × 24 cm). Six cuttings were transferred to each container, with six containers for each medium type. Hydroponic and agar (0.1% *w/v*) media were dissolved in a nutrient solution containing 4-mM NH_4_NO_3_, 0.6-mM NaH_2_PO_4_, 0.6-mM KCl, 0.35-mM CaCl_2_, 0.25-mM MgSO_4_, 10-μM FeSO_4_, 20-μM H_3_BO_3_, 2-μM MnCl_2_, 2-μM ZnSO_4_, 2-μM CuSO_4_, and 2-μM Na_2_MoO_4_, at a pH between 5.5 and 6.0 [22]. Plants were cultivated in a phytotron under the same conditions as mentioned above. Cuttings were transferred to fresh medium every 2 weeks. Plants were grown for about 5 months on hydroponic or agar media, by which time the adventitious roots had formed and were used to examine the source of O_2_. Thereafter, the water level was raised by 6 cm from the stem base in the same medium conditions. Plants were grown for about 3 months after water level elevation, by which time new adventitious roots had formed near the raised water level. These newly developed adventitious roots were also used to examine the entry portion of atmospheric O_2_ in the stem, which was based on the elevated water level.

### 4.2. O_2_ Microelectrode Measurements

For each experiment, cuttings were transferred into a container filled with deoxygenated solid agar (1.5% *w/v*) and kept at a constant room temperature (25–27 °C) under a photosynthetic photon flux density (PPFD) of about 100 μmol m^−2^ s^−1^ provided by fluorescent tubes (FL20SBR, NEC, Tokyo, Japan). Cuttings were fixed in the container by solid agar, maintaining the water level similar to that when the cuttings were grown in hydroponic or agar media. The target adventitious roots were fixed on the surface of the solid agar and covered with deoxygenated solid gelrite (2% *w/v*), about 1-cm-thick, to ensure a clear view under a microscope (Figure 1). Experiments were conducted both before and after the water level was raised. Using a micromanipulator, a Clark-type O_2_ microelectrode was positioned in the cortex (primary aerenchyma), about 100 μm from the surface of the adventitious roots that developed 10 cm from the root–shoot junction. The positioning of the microelectrode inside the root cortex was aided by a microscope (SZX7, Olympus, Tokyo, Japan) and adjusted using a boom stand (SZ2-STU2, Olympus, Tokyo, Japan).

### 4.3. Effect of Light Conditions on the Root Oxygen Concentration

We investigated the changes in O_2_ concentration in roots under light and dark conditions to confirm whether the O_2_ produced by photosynthesis affects the O_2_ concentration in roots. Changes in the O_2_ concentrations in roots were measured when the fluorescent light (PPFD about 100 μmol m^−2^ s^−1^) was switched on and off with the O_2_ microelectrode inserted into the root. To avoid the effects of natural light coming through windows, experiments were conducted after sunset. 

The light condition was controlled during an injection of air or nitrogen (N_2_) into the stem 0–3 cm above the water level. Air and N_2_ were alternately injected through the cuvette at intervals of 30 min in the stem 0–3 cm above the water level. Then, the change in the root pO_2_ was measured at the cortex, 10 cm from the root–shoot junction (Figure 1).

### 4.4. Function of Secondary Aerenchyma Developed in the Stem as an Oxygen Pathway 

Changes in root pO_2_ before and after the removal of secondary aerenchyma in the stem were observed. Five months after hypoxic treatment, about 1 cm of bark length, including the secondary aerenchyma, was removed around the circumference of the stem, 2 cm below the water level. Then, air or N_2_ was injected through the cuvette into the stem 0–3 cm above the water level. Next, the change in root pO_2_ was measured at the cortex, 10 cm from the root–shoot junction (Figure 2). 

### 4.5. Entry Portion of Atmospheric Oxygen in the Stem

The entry portion of atmospheric O_2_ into the adventitious roots was investigated by measuring the change in pO_2_ at the intact adventitious roots while changing the position of the stem exposed to either air or N_2_. Air or N_2_ was injected through a 3-cm cuvette and plastic bag to control the O_2_ condition of the stem and leaf. The positions of the cuvette and plastic bag were changed on the stem at four points above the water level: 0–3 cm, 3–6 cm, 6–9 cm, and the leaf level. Cuvettes and transparent plastic bags enclosed the stems and leaves, respectively; they were fixed with paraffin film to the stem. The air and N_2_ injections lasted 30 min each; hence, the stem and leaf were both enclosed for 1 h in the cuvette and in the sealed plastic bag, respectively. Then, the change in the root pO_2_ was measured at the cortex of the adventitious root at 10 cm from the root–shoot junction (Figure 3). The inlet and outlet were installed at the upper and lower ends of the cuvette or plastic bag, respectively. The inflow of the gas into the cuvette was limited to the inlet. Air and N_2_ were supplied by connecting a silicone tube with the cuvette and plastic bag.

### 4.6. Entry Portion of Atmospheric oxygen into the Stem after Water Level Elevation

After the water level was raised by 6 cm above the previous water surface level, new adventitious roots (upper position) developed from the submerged stem near the elevated water surface. They began to develop about 1 to 2 weeks after the water level was elevated. The experiments were performed 3 months after raising the water level.

### 4.7. Upper (New) Adventitious Roots

To investigate the entry portion of atmospheric O_2_ into the newly developed adventitious roots after raising the water level, the changes in pO_2_ in the intact newly developed adventitious roots were measured while changing the position of the stem exposed to air or N_2_. The root system was fixed with deoxygenated solid agar (1.5 % *w/v*). The position of the cuvette was changed on the stem at four places, according to the raised water level: 0–3 cm, 3–6 cm, and 6–9 cm above the water level and the leaf. N_2_ was injected for 30 min through the cuvette into the stem and for 1 h through a plastic bag-sealed leaf. Then, the change in the root pO_2_ was measured at the cortex of the adventitious root, 10 cm from the root–shoot junction (Figure 8 and Figure 9).

### 4.8. Lower (Old) Adventitious Roots 

To investigate the effect of water elevation on the entry portion of O_2_ into the lower adventitious roots—that is, the old adventitious roots that were developed before the water level elevation—the changes in the pO_2_ were measured in the intact lower adventitious roots while 0–3 cm of stem based on the elevated water level was exposed to air or N_2_. The root system was fixed with deoxygenated solid agar (1.5% *w/v*), and the agar around the stem, positioned at 0–3 cm above the prior water level, was removed. The upper adventitious roots and stem were embedded in solid agar. Then, air or N_2_ was injected into the cuvette, where the agar was removed, and the change in the pO_2_ of the lower adventitious roots was observed. The change in the root pO_2_ was measured in the cortex of the adventitious root at 10 cm from the root–shoot junction (Figure 8 and Figure 9).

## 5. Conclusions

*Syzygium kunstleri* adapts to hypoxic conditions by developing new adventitious roots. The entry portion of atmospheric oxygen was determined as the portion of the stem 0–3 cm above the water surface. The secondary aerenchyma in the stem functions as an O_2_ transport pathway from the stem to the roots. However, the internal O_2_ transport in the adventitious roots does not depend on O_2_ generated by photosynthesis. Change in the water level results in the generation of new adventitious roots from the submerged stem near the elevated water surface, and the stem portion, 0–3 cm above the elevated water surface, is the entry portion of atmospheric oxygen for the newly developed root. It was clarified that atmospheric oxygen near the water surface moves to the adventitious roots developed from the submerged stem near the water surface through the secondary aerenchyma formed in the stem. The obtained results could be useful for understanding the environmental adaptation mechanisms to flooding, especially in woody plants.
